# Prescription Medication Expenditures for Patients With Diabetes in the United States: 2012–2021

**DOI:** 10.1111/1753-0407.70106

**Published:** 2025-07-22

**Authors:** Shanshan Li, Shaoxi Pan, Nan Xiao, Shaoxiang Jiang, Gorden G. Liu, Beini Lyu

**Affiliations:** ^1^ Department of Global Health School of Public Health, Peking University Beijing China; ^2^ Institute for Global Health and Development, Peking University Beijing China; ^3^ China Center for Health Economic Research, Peking University Beijing China; ^4^ School of Public Health, the Key Laboratory of Environmental Pollution Monitoring and Disease Control, Ministry of Education Guizhou Medical University Guiyang China; ^5^ National School of Development, Peking University Beijing China

**Keywords:** glucose‐lowering drugs, Medicare, medication expenditures, non‐glucose‐lowering drugs, private insurance

## Abstract

Glucose‐lowering medication expenditures per user by different payers among patients with diabetes.

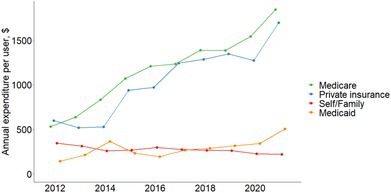

1

**Summary**

- Annual prescription medication expenditures per person increased from $3932 in 2012 to $6397 in 2021 for patients with diabetes, driven primarily by increased spending on insulin and non‐insulin glucose‐lowering drugs.
- The majority of prescription medication expenditures were borne by Medicare and private insurance.
- These findings emphasize the need for policies to control the rising costs of medications, especially glucose‐lowering medications.

To the Editor,

Diabetes imposes an enormous medical and economic burden on individuals and society, particularly regarding prescription medications [1, 2]. As the number and complexity of medications grow markedly [3], a systematic examination of medication expenditures is essential. However, previous studies on medication expenditures among patients with diabetes have mainly focused on glucose‐lowering medications, and few have incorporated non‐glucose‐lowering medications. Such an analysis is needed to provide a comprehensive understanding of medication expenditures, to reveal opportunities to alleviate the economic burdens, and to guide resource allocation. We aimed to examine overall prescription medication expenditures, as well as by therapeutic class and payment sources for patients with diabetes in the United States.

## 1 Methods

We utilized data from the Medical Expenditure Panel Survey (MEPS), a nationally representative survey in the United States [4]. We included patients ≥ 18 years and reported being diagnosed with diabetes (both Types 1 and 2) from 2012 to 2021. Prescription medication expenditures were the amount that pharmacy received during the survey year for all prescription medication [5]. Expenditures were categorized by therapeutic class and payment sources (including Medicare, Medicaid, private insurance, and out‐of‐pocket [OOP] payments). We calculated annual medication expenditures per person, expenditures per user, overall and by payment sources, expenditures among different insurance beneficiaries, and the proportion of expenditures attributed to a certain therapeutic class. Please see detailed methods in Item S1.

## 2 Results

The study included 30 146 patients with diabetes (mean [95% confidence interval] age 62.4 [54.0–72.0] years, Figure S1 and Table S1). Annual prescription medication expenditures per person increased from $3932 in 2012 to $6397 in 2021, with Medicare being the largest source of payment ($1468 in 2012 to $2948 in 2021), followed by private insurance ($1090 in 2012 to $1951 in 2021) and Medicaid ($313 in 2012 to $877 in 2021, Figure S2), while OOP cost decreased from $782 in 2012 to $415 in 2021. Similar patterns were found among insurance beneficiaries (Figure S3). Expenditures were highest among Medicare beneficiaries ($3344 in 2012 to $6170 in 2021), followed by those covered by private insurance and Medicaid.

Glucose‐lowering medications contributed the largest proportion of expenditures (36.9% in 2012 to 58.6% in 2021, Figure 1), with a substantial increase attributed to non‐insulin glucose‐lowering medications (24.8% in 2012 to 45.3% in 2021). The proportion attributed to insulin remained stable (12.1% in 2012 to 13.3% in 2021). Expenditures attributed to non‐glucose‐lowering medications decreased over time and accounted for 41.4% in 2021.

**FIGURE 1:**
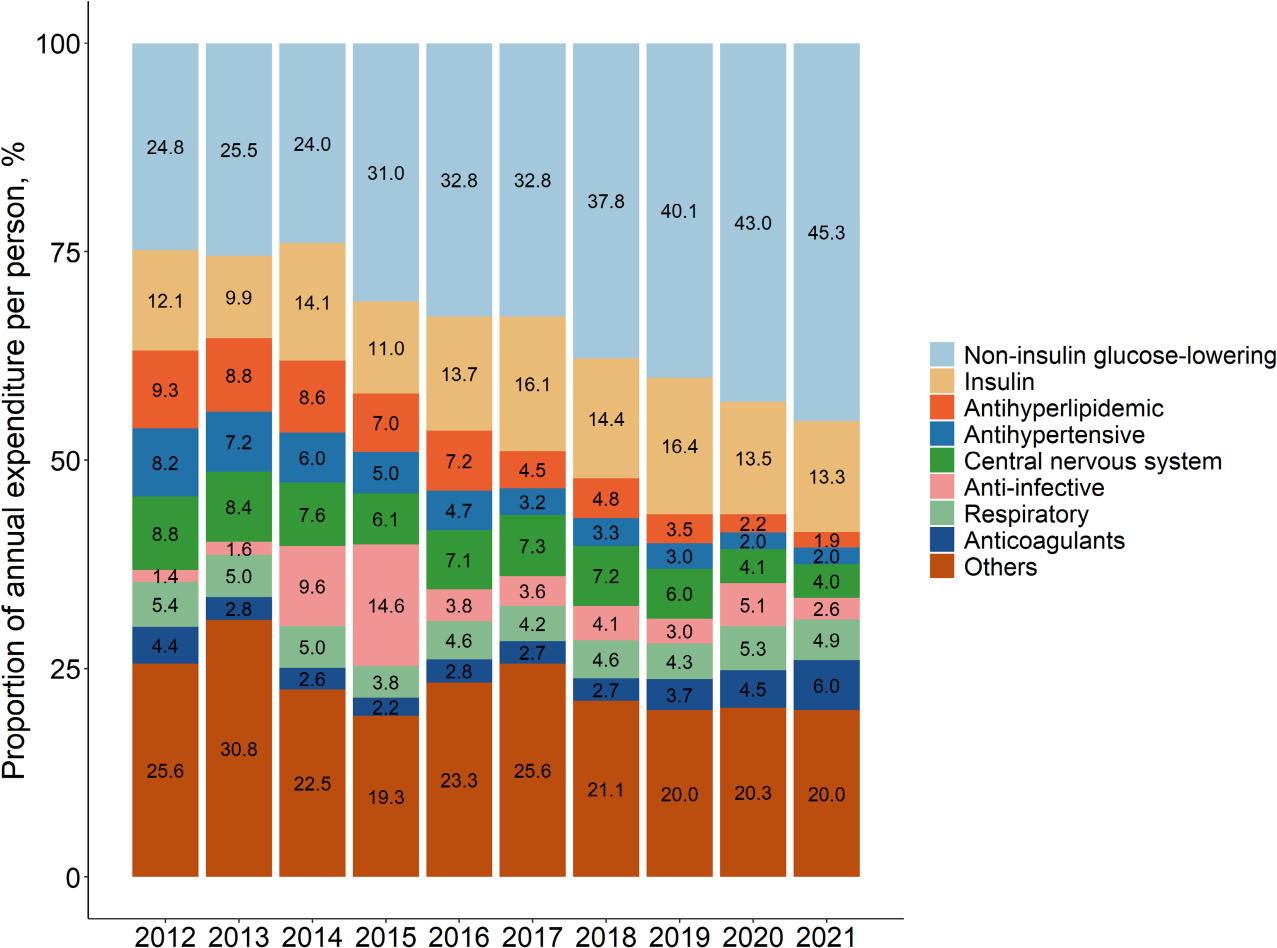
Proportion of annual medication expenditures per person attributed to different therapeutic classes.

**FIGURE 2:**
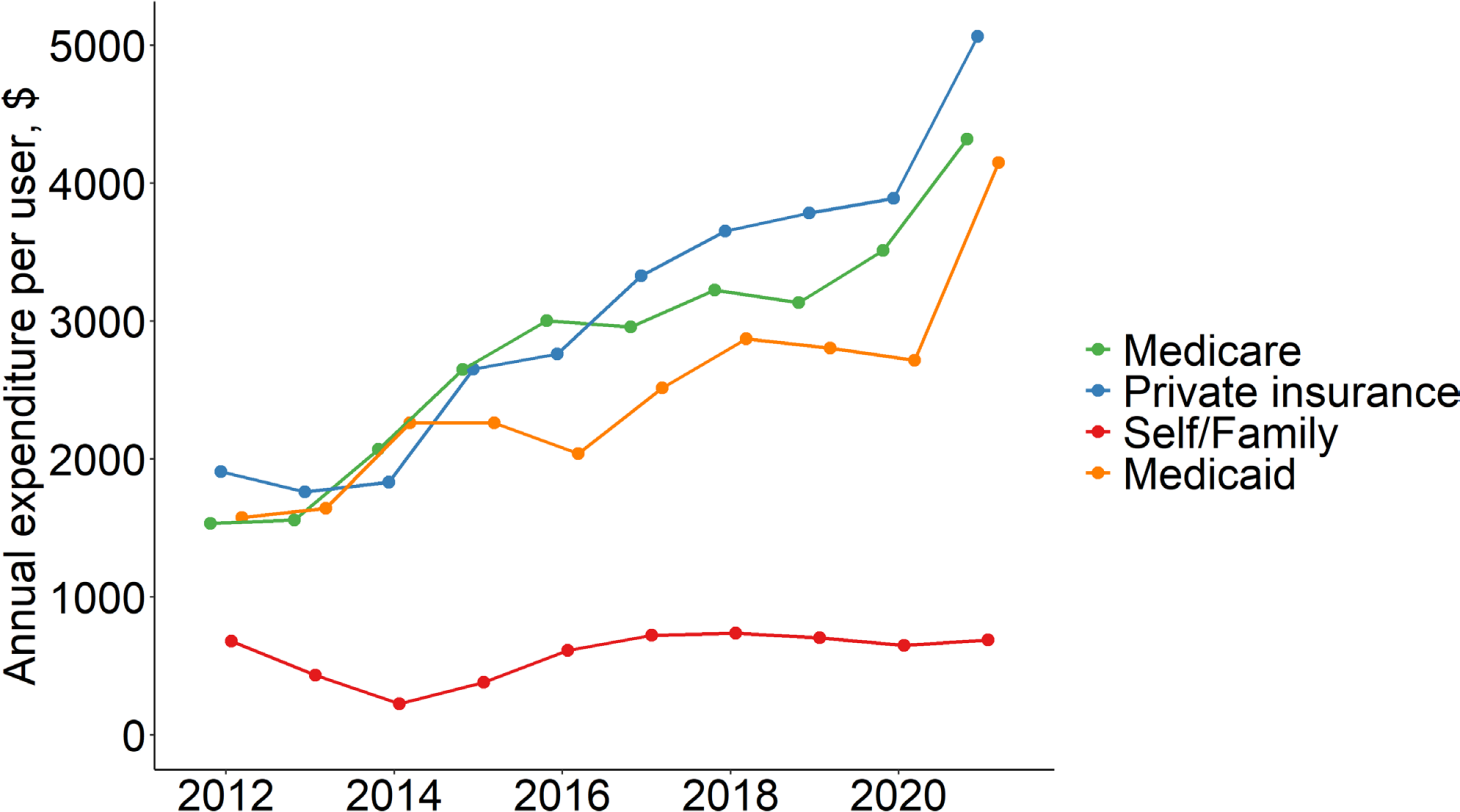
Glucose‐lowering medication expenditures per user by different payers among patients with diabetes.

For annual cost per user, the cost was highest for glucose‐lowering medications (from $1701 in 2012 to $4393 in 2021), with a substantial increase for insulin ($3301 to $6618, a 100.5% increase) and non‐insulin drugs ($1345 to $3765, a 180.0% increase). Medicare and private insurance were the main payment sources, and both increased substantially over time (Figure 2). We found similar patterns among various insurance beneficiaries (Figure S4).

## 3 Comment

In summary, prescription medication expenditures increased substantially over the past decade among patients with diabetes, with the majority of expenditures attributed to glucose‐lowering medication and borne by Medicare and private insurance.

Consistent with studies in the United States and outside of the United States [6, 7, 8, 9, 10], medication costs increased among patients with diabetes, especially for glucose‐lowering medications. The increasing use of newer and more expensive medications such as sodium‐glucose cotransporter‐2 inhibitors and greater use of costly insulin analogs rather than cheaper human insulin likely contributes to this surge [10, 11]. Non‐glucose‐lowering medications also constituted a substantial portion of medication expenditures. The use of these medications, such as central nervous system, anti‐infective, and respiratory medications, is prevalent in patients with diabetes, consistent with the increasing burden of comorbidities among these patients [3, 12, 13].

We found decreasing OOP medication expenditures over time. Policy reforms such as the Medicare Part D, the Affordable Care Act (ACA), and Medicaid expansion could have contributed to OOP costs decrease [14, 15]. In contrast, medication expenditures borne by insurance increased substantially, especially among beneficiaries. The untransparent discounts and rebates between the pharmacy and insurance in the United States likely complicate the calculation of costs [16, 17, 18]. Nonetheless, the increasing spending by insurance may lead to higher premiums and increase patients' financial burden [19]. Policies such as the Inflation Reduction Act allow price negotiation and rebates for several non‐insulin glucose‐lowering medications in Medicare and cap Medicare Parts B and D insulin cost‐sharing [20, 21]. This is expected to reduce Medicare prescription drug spending [22].

Our study had several limitations. First, the diagnosis of diabetes and comorbidities were based on self‐report. Second, over‐the‐counter medications were not accounted for in the MEPS. Third, expenditures were measured as the total amount paid by insurers and patients, and it might differ from the amount received by manufacturers due to discounts and rebates.

In summary, prescription medication expenditure increased substantially for patients with diabetes, especially for glucose‐lowering medications. With ongoing policies to reduce medication costs, future studies are needed to evaluate policy impact on medication costs and patient outcomes among patients with diabetes.

## Author Contributions

S. Li, S. Pan, N. Xiao, S. Jiang, G.G. Liu, and B. Lyu were involved in the conception, design, and conduct of the study and the analysis. All authors were involved in the interpretation of the results. S. Li and B. Lyu wrote the first draft of the manuscript, and all authors edited, reviewed, and approved the final version of the manuscript. S. Li and B. Lyu are the guarantors of this work and, as such, had full access to all the data in the study and take responsibility for the integrity of the data and the accuracy of the data analysis.

## Disclosure

The authors have nothing to report.

## Conflicts of Interest

The authors declare no conflicts of interest.

## Supporting information


Data S1.

## Data Availability

The MEPS data are publicly available at https://meps.ahrq.gov/mepsweb/.
