# Correction to “Prescription Medication Expenditures for Patients With Diabetes in the United States: 2012–2021”

**DOI:** 10.1111/1753-0407.70158

**Published:** 2025-10-08

S. Li, S. Pan, N. Xiao, S. Jiang, G. G. Liu, and B. Lyu, “Prescription Medication Expenditures for Patients With Diabetes in the United States: 2012–2021,” *Journal of Diabetes* 17, no. 7 (2025): e70106, https://doi.org/10.1111/1753‐0407.70106.

In the published version of the article, Figure 2 in the main text was incorrectly linked to Figure S4 (Supporting Information). The correct Figure 2 should correspond to the Graphical Abstract Image, as intended and originally submitted.

Correct Figure 2 is shown here:

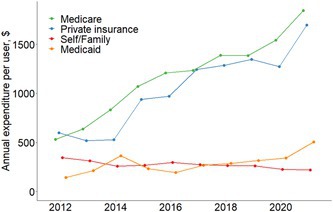

We apologize for this error.

