# Supplementary material for: Prescription Medication Expenditures for Patients With Diabetes in the United States: 2012–2021
Source: J Diabetes. 2025 Jul 22;17(7):e70106. doi: 10.1111/1753-0407.70106 (PMC12280806; doi:10.1111/1753-0407.70106)
Supplement: Supplementary file 1 — Data S1. [file JDB-17-e70106-s001.docx]

# Prescription Medication Expenditures for Patients with Diabetes in the United States: 2012-2021

Shanshan Li^1,2.3^ | Shaoxi Pan^3,4^ | Nan Xiao^2,5^ | Shaoxiang Jiang^2^ | Gorden G. Liu^2,3,5^ | Beini Lyu^2^

^1^ Department of Global Health, School of Public Health, Peking University, Beijing, China

^2^ Institute for Global Health and Development, Peking University, Beijing, China

^3^ China Center for Health Economic Research, Peking University, Beijing, China

^4^ School of Public Health, the key Laboratory of Environmental Pollution Monitoring and Disease Control, Ministry of Education, Guizhou Medical University, Guiyang

^5^ National School of Development, Peking University, Beijing, China

Table of Contents

Item S1 Detailed methodology description

Table S1. Characteristics of patients with diabetes

Figure S1. Flowchart for study population. MEPS, Medical Expenditure Panel Survey

Figure S2. Annual medication expenditures per person overall and by sources of payment among the entire patient population. (A) Overall, (B) By sources of payment

Figure S3. Annual medication expenditures per person among insurance beneficiaries

Figure S4. Glucose-lowering medication expenditures per user among insurance beneficiaries

Item S1 Detailed methodology description

**Data Source and Study Population**

The Medical Expenditure Panel Survey (MEPS) is a nationally representative survey conducted by the Agency for Healthcare Research and Quality (AHRQ). The survey focuses on the civilian population of the United States and their healthcare providers, including physicians, hospitals, and pharmacies. The MEPS collects information on healthcare utilization, expenditures, and the health status of each household member. Our study utilized the household full year consolidated data file, the medical condition file, and the prescribed medicines files from the Household Component. Our study included individuals aged 18 or older who reported being diagnosed with diabetes (both type 1 and type 2) by healthcare professionals from 2012 to 2021.

**Prescription Medication Expenditures**

MEPS respondents self-reported the names of any prescribed medications procured at any pharmacy, including mail-order or on-line order, acquired by themselves or their family members during the calendar year. Following written authorization to access payment data, information about the date of prescription fulfillment, national drug code, medication name, medication strength, quantity, payment amount, and sources of payment were obtained from pharmacies.^1^ Medications were categorized into therapeutic classes according to the Multum Lexicon classification scheme. The scheme is comprehensive and includes all prescription and some nonprescription medications available in the US drug market. The Multum’s Lexicon scheme provides a 3-level nested category system that assigns a therapeutic classification to each drug and each ingredient of the drug. For example, the code for naproxen is central nervous system agents (level 1), analgesics (level 2) and nonsteroidal anti-inflammatory agents (level 3). ^2^

Annual prescription medication expenditures were measured as the total payments received by pharmacy during the survey year and included expenditures for all prescription medication. These expenditures included both out-of-pocket (OOP) payments and payments made by private and public health insurances, categorized by therapeutic classes and by sources of payment. Payment sources included Medicare, Medicaid, private insurance, and OOP (self/family) payments. Specifically, Medicare is funded through federal sources and primarily serves people over the age of 65 and younger individuals who qualify for Social Security Disability Insurance, such as people with end-stage kidney disease; Medicaid is jointly funded by the federal government and the states and serves low-income individuals and families and people with disabilities; and private insurance are available to individuals, families, and companies offering insurance to their employees, with varying types of coverage.

To assess the economic burden of medication expenditures, we first calculated medication expenditure per person across the whole patient population (e.g., dividing the total medication expenditures by the total number of patients with diabetes). We then detailed the total expenditures, breaking them down by sources of payment and therapeutic classes. Proportion of expenditures attributed to a certain therapeutic class was calculated by dividing the expenditures of medications in a specific therapeutic class by the total medication expenditures. Recognizing the variability in user numbers for specific medications, we then calculated expenditures per user to better reflect the individual economic impact (e.g., dividing the expenditures of a specific medication by the number of patients who used that medication). The expenditure on medications across different insurance categories may be influenced by both the number of patients covered by each type of insurance and the average expenditure per beneficiary. To distinguish the effects of these two factors, we have conducted further analyses to estimate the mean expenditures among beneficiaries of each insurance category.

**Characteristics**

We included the following characteristics: age (categorized as 18-44 years, 45-64 years, or ≥65 years), sex (male or female), race and ethnicity (Hispanic, White, Black, or Others), health insurance status (uninsured, public insurance only, or private insurance), marital status (married or not married), education (less than high school, high school/general educational development, or some college degree), income, and comorbidity burden. Income was categorized as poverty (<100% of the federal poverty level [FPL]), near poverty (100% - 125% FPL), low income (125% - 200% FPL), moderate income (200% - 400% FPL), and high income (≥400% FPL). Comorbidity burden was measured by Charlson Comorbidity Index (CCI) and categorized as 0-1 or ≥2.^3^

**Statistical Analyses**

To account for unequal sampling probability and nonresponse, all analyses incorporated the MEPS sampling weights provided by the AHRQ to generate representative estimates. We employed Taylor-series linearization methods to estimate standard errors and reported 95% confidence intervals (CIs) for population estimates. Continuous variables were presented as means with corresponding 95% CIs and categorical variables were presented as percentages with corresponding 95% CIs. OOP expenditures were adjusted to 2021 dollars using the Consumer Price Index for medical care and other expenditures were adjusted to 2021 dollars using the price index for prescription drugs. All analyses were conducted using Stata, version 17 (StataCorp, College Station, TX) and R (www.R-project.org/). Statistical significance was defined as a two-sided p-value less than 0.05.

**References**

1.MEPS HC-220A: 2020 Prescribed Medicines File. Accessed May 21, 2024. <https://meps.ahrq.gov/data_stats/download_data/pufs/h220a/h220adoc.shtml#Data25>

2.National Center for Health Statistics, Centers for Disease Control and Prevention (CDC). RXQ_DRUG. Accessed December 31, 2024. <https://wwwn.cdc.gov/Nchs/Data/Nhanes/Public/1988/DataFiles/RXQ_DRUG.htm#Appendix_3:_Multum_Lexicon_Therapeutic_Classification_Scheme>

3.Charlson ME, Pompei P, Ales KL, MacKenzie CR. A new method of classifying prognostic comorbidity in longitudinal studies: Development and validation. *Journal of Chronic Diseases*. 1987;40(5):373-383. doi:[10.1016/0021-9681(87)90171-8](https://doi.org/10.1016/0021-9681(87)90171-8)

_

**Table S1. Characteristics of patients with diabetes**

| **Characteristics*** | **2012**  **N = 2492** | **2013**  **N =**  **3233** | **2014**  **N =**  **3166** | **2015**  **N = 3151** | **2016**  **N = 3854** | **2017**  **N = 3241** | **2018**  **N = 2657** | **2019**  **N = 2558** | **2020**  **N = 2797** | **2021**  **N = 2997** | **P value** ^†^ |
| --- | --- | --- | --- | --- | --- | --- | --- | --- | --- | --- | --- |
| **Age, years** | 61.5  (53.0-72.0) | 62.5  (53.0-72.0) | 62.6  (53.0-73.0) | 62.0  (53.0-72.0) | 62.2  (53.0-72.0) | 62.4  (54.0-71.0) | 62.5  (54.0-73.0) | 62.5  (54.0-73.0) | 62.8  (54.0-73.0) | 62.6  (54.0-73.0) | 0.19 |
| **Age group, years** |  |  |  |  |  |  |  |  |  |  | 0.005 |
| 20-44 | 12.1  (10.4-13.8) | 10.7  (9.3-12.0) | 10.2  (8.8-11.6) | 9.7  (8.4-10.9) | 9.5  (8.3-10.7) | 10.0  (8.6-11.3) | 11.2  (9.7-12.8) | 11.4  (9.7-13.1) | 10.5  (8.9-12.2) | 11.4  (10.0-12.9) |  |
| 45-64 | 44.2  (41.1-47.3) | 42.4  (40.1-44.7) | 42.0  (39.4-44.5) | 45.2  (43.1-47.2) | 45.2  (43.2-47.2) | 43.8  (41.5-46.0) | 40.6  (38.1-43.1) | 39.6  (37.3-41.9) | 40.5  (38.2-42.8) | 39.3  (36.8-41.8) |  |
| ≥65 | 43.7  (40.5-46.9) | 46.9  (44.3-49.5) | 47.9  (45.2-50.6) | 45.2  (43.0-47.4) | 45.3  (43.2-47.4) | 46.3  (44.0-48.6) | 48.2  (45.5-50.8) | 49.0  (46.5-51.5) | 49.0  (46.6-51.3) | 49.3  (46.8-51.7) |  |
| **Sex, Female** | 49.3  (47.0-51.7) | 51.3  (49.2-53.5) | 52.5  (50.1-54.9) | 50.7  (48.7-52.8) | 50.0  (48.5-51.4) | 49.3  (47.4-51.1) | 48.4  (46.1-50.6) | 48.6  (46.7-50.5) | 49.5  (47.6-51.4) | 48.9  (46.7-51.2) | 0.10 |
| **Race/Ethnicity** |  |  |  |  |  |  |  |  |  |  | 0.39 |
| Hispanic | 15.6  (12.9-18.2) | 14.3  (12.5-16.1) | 14.2  (12.3-16.2) | 15.2  (13.5-17.0) | 15.7  (14.0-17.3) | 15.3  (13.7-17.0) | 15.0  (12.5-17.5) | 16.0  (13.5-18.5) | 16.9  (14.2-19.6) | 15.5  (13.0-18.1) |  |
| Non-Hispanic Black | 15.3  (13.2-17.5) | 14.9  (13.2-16.6) | 15.0  (13.2-16.8) | 15.9  (14.0-17.9) | 14.9  (13.5-16.4) | 14.6  (13.1-16.1) | 14.8  (12.7-16.9) | 14.7  (12.6-16.8) | 14.4  (12.4-16.4) | 14.9  (12.8-17.1) |  |
| Non-Hispanic White | 61.4  (58.0-64.7) | 62.7  (60.2-65.2) | 62.5  (59.8-65.1) | 60.8  (58.0-63.5) | 61.5  (59.1-63.9) | 61.4  (59.0-63.9) | 61.4  (58.4-64.5) | 59.4  (56.3-62.4) | 58.8  (55.5-62) | 58.6  (55.4-61.9) |  |
| Others | 7.7  (5.8-9.6) | 8.1  (6.7-9.4) | 8.3  (6.6-10.0) | 8.0  (6.4-9.6) | 7.9  (6.4-9.4) | 8.6  (7.0-10.2) | 8.8  (7.2-10.4) | 9.9  (8.0-11.8) | 9.9  (8.0-11.8) | 10.9  (8.6-13.2) |  |
| **Marital status, Married** | 56.1  (53.7-58.6) | 55.2  (52.5-57.9) | 56.2  (53.8-58.7) | 59.2  (56.9-61.5) | 57.8  (55.6-59.9) | 53.1  (50.9-55.4) | 54.3  (51.9-56.7) | 53.7  (51.4-56.0) | 53.0  (50.5-55.4) | 52.7  (50.3-55.1) | <0.001 |
| **Education level** |  |  |  |  |  |  |  |  |  |  | <0.001 |
| Less than high school | 21.8  (19.1-24.5) | 20.1  (18.4-21.8) | 18.5  (16.9-20.2) | 20.8  (18.7-22.9) | 19.8  (18.1-21.4) | 17.8  (16.0-19.6) | 18.6  (16.4-20.8) | 19.7  (17.6-21.9) | 17.6  (15.5-19.7) | 15.5  (13.6-17.5) |  |
| High school/General educational development | 61.1  (57.6-64.6) | 61.3  (58.9-63.6) | 62.4  (60.2-64.6) | 58.1  (55.1-61.1) | 58.7  (56.5-60.9) | 57.7  (55.2-60.2) | 55.8  (53.3-58.3) | 55.2  (52.5-57.8) | 53.5  (50.6-56.3) | 54.7  (52.1-57.4) |  |
| Some college | 17.1  (14.3-19.8) | 18.6  (16.5-20.8) | 19.1  (17.0-21.2) | 21.1  (18.6-23.7) | 21.6  (19.4-23.7) | 24.5  (22.2-26.8) | 25.6  (23.3-27.8) | 25.1  (22.7-27.5) | 28.9  (26.4-31.5) | 29.7  (27.2-32.3) |  |
| **Insurance status** |  |  |  |  |  |  |  |  |  |  | <0.001 |
| Any private | 57.4  (54.4-60.4) | 57.0  (54.5-59.5) | 56.6  (54.1-59.1) | 57.8  (55.6-60.1) | 58.4  (56.5-60.2) | 58.1  (55.9-60.2) | 55.0  (52.2-57.8) | 51.9  (49.6-54.2) | 51.8  (49.3-54.3) | 52.9  (50.3-55.6) |  |
| Public only | 35.0  (32.3-37.8) | 35.7  (33.3-38.0) | 37.7  (35.4-40.1) | 36.5  (34.5-38.6) | 36.6  (34.8-38.4) | 38.4  (36.4-40.5) | 42.0  (39.4-44.6) | 45.0  (42.8-47.3) | 45.0  (42.5-47.5) | 43.7  (41.1-46.2) |  |
| No insurance | 7.6  (6.3-8.8) | 7.3  (6.2-8.4) | 5.7  (4.7-6.7) | 5.6  (4.6-6.7) | 5.0  (4.1-5.9) | 3.5  (2.8-4.2) | 3.0  (2.3-3.7) | 3.0  (2.2-3.9) | 3.2  (2.4-4.0) | 3.4  (2.5-4.3) |  |
| **Income** |  |  |  |  |  |  |  |  |  |  | 0.019 |
| Poverty | 14.5  (12.3-16.7) | 14.5  (12.8-16.2) | 16.2  (14.5-17.8) | 13.6  (12.2-15.0) | 13.9  (12.5-15.3) | 15.1  (13.6-16.5) | 14.4  (12.8-16.1) | 15.5  (13.9-17.1) | 15.3  (13.7-17.0) | 14.4  (12.7-16.1) |  |
| Near-poor | 7.0  (5.6-8.5) | 6.0  (4.9-7.0) | 6.1  (5.1-7.2) | 5.6  (4.7-6.5) | 6.4  (5.5-7.3) | 5.8  (4.8-6.8) | 6.4  (5.1-7.6) | 5.2  (4.1-6.3) | 5.3  (4.4-6.3) | 6.1  (5.0-7.1) |  |
| Low income | 17.7  (15.7-19.7) | 16.5  (14.9-18.1) | 16.6  (14.9-18.2) | 17.7  (16.0-19.4) | 16.8  (15.3-18.3) | 16.1  (14.5-17.8) | 16.2  (14.3-18.1) | 15.4  (13.5-17.3) | 14.8  (13.3-16.4) | 14.5  (12.8-16.2) |  |
| Moderate income | 30.4  (27.9-33.0) | 31.3  (29.3-33.2) | 27.1  (25.0-29.2) | 29.5  (27.2-31.9) | 26.5  (24.5-28.5) | 28.3  (26.3-30.3) | 28.2  (25.9-30.6) | 29.7  (27.7-31.7) | 27.2  (25.0-29.4) | 28.9  (26.8-31.1) |  |
| High income | 30.3  (27.5-33.2) | 31.8  (29.3-34.2) | 34.0  (31.3-36.8) | 33.6  (31.0-36.2) | 36.4  (34.0-38.8) | 34.7  (32.2-37.2) | 34.7  (31.8-37.6) | 34.2  (31.6-36.8) | 37.3  (34.5-40.1) | 36.1  (33.7-38.6) |  |
| **Charlson comorbidity index** |  |  |  |  |  |  |  |  |  |  | 0.014 |
| 0-1 | 14.4  (12.9-16.2) | 13.2  (11.9-14.6) | 11.7  (10.4-13.2) | 12.7  (11.3-14.3) | 11.8  (10.5-13.2) | 12.0  (10.8-13.4) | 14.4  (13.0-16.1) | 15.5  (13.6-17.6) | 14.4  (13.0-16.0) | 14.5  (12.0-16.3) |  |
| ≥2 | 85.6  (83.9-87.2) | 86.8  (85.5-88.2) | 88.3  (86.9-89.7) | 87.3  (85.8-88.8) | 88.2  (86.9-89.5) | 88.0  (86.7-89.3) | 85.6  (84.0-87.1) | 84.5  (82.6-86.5) | 85.6  (84.1-87.1) | 85.5  (83.8-87.1) |  |

* Data presented were weighted to be nationally representative. Continuous variables were presented as means (95% confidence intervals, 95% CIs) and categorical variables were presented as percentages (95% CIs).

^†^ Chi-squared test with Rao & Scott’s second-order correctio.


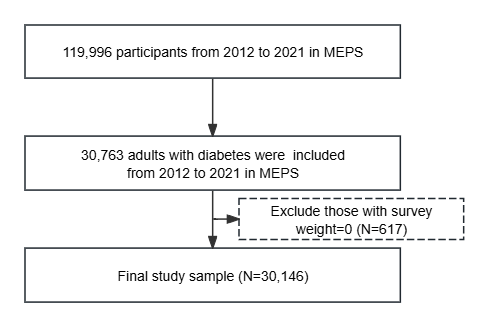


**Figure S1. Flowchart for study population. MEPS, Medical Expenditure Panel Survey**


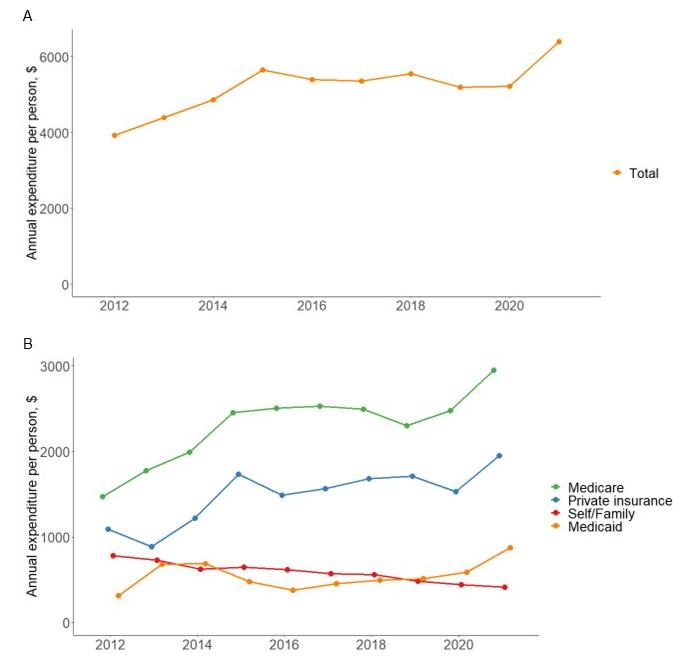


**Figure S2.** **Annual medication expenditures per person overall and by sources of payment among the entire patient population. (A) Overall, (B) By sources of payment**


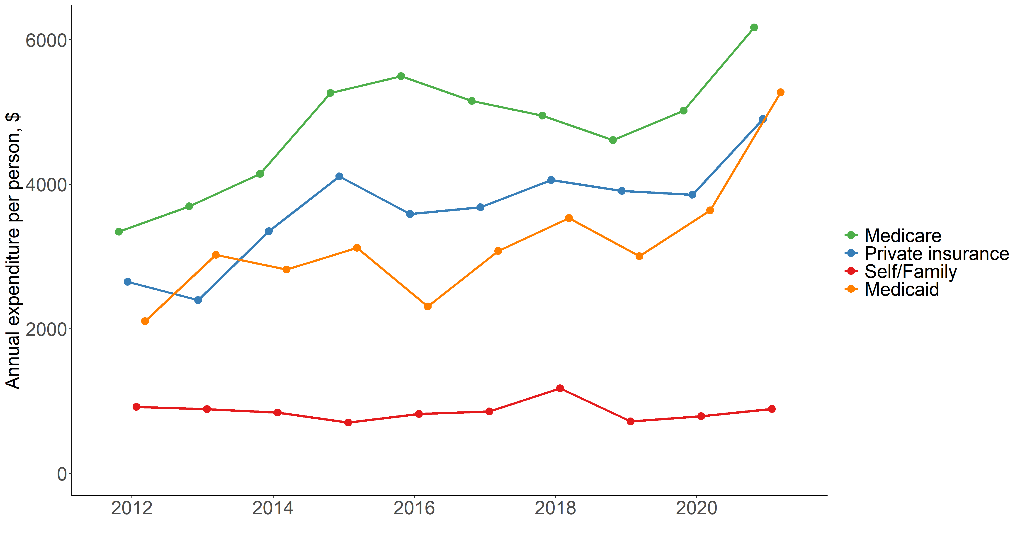


**Figure S3. Annual medication expenditures per person among insurance beneficiaries**


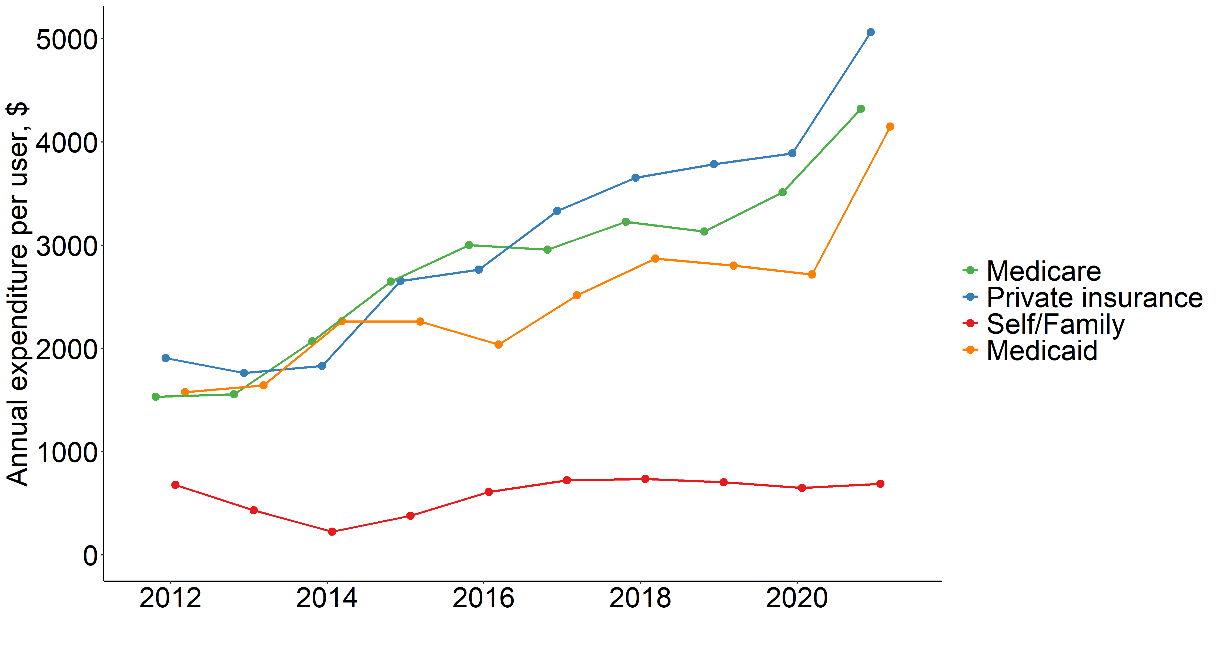


**Figure S4. Glucose-lowering medication expenditures per user among insurance beneficiaries**
